# A combined association mapping and t-test analysis of SNP loci and candidate genes involving in resistance to low nitrogen traits by a wheat mutant population

**DOI:** 10.1371/journal.pone.0211492

**Published:** 2019-01-30

**Authors:** Hongchun Xiong, Huijun Guo, Chunyun Zhou, Xiaotong Guo, Yongdun Xie, Linshu Zhao, Jiayu Gu, Shirong Zhao, Yuping Ding, Luxiang Liu

**Affiliations:** 1 Institute of Crop Sciences, Chinese Academy of Agricultural Sciences/National Engineering Laboratory for Crop Molecular Breeding, National Center of Space Mutagenesis for Crop Improvement, Beijing, China; 2 College of Agriculture, Ludong University, Yantai, China; Murdoch University, AUSTRALIA

## Abstract

Crop productivity is highly dependent on the application of N fertilizers, but ever-increasing N application is causing serious environmental impacts. To facilitate the development of new wheat cultivars that can thrive in low N growth conditions, key loci and genes associated with wheat responses to low N must be identified. In this GWAS and *t*-test study of 190 M_6_ mutant wheat lines (Jing 411-derived) based on genotype data from the wheat 660k SNP array, we identified a total of 221 significant SNPs associated four seedling phenotypic traits that have been implicated in resistance to low N: relative root length, relative shoot length, relative root weight, and relative shoot weight. Notably, we detected large numbers of significantly associated SNP in what appear to be genomic ‘hotspots’ for resistance to low N on chromosomes 2A and 6B, strongly suggesting that these regions are functionally related to the resistance phenotypes that we observed in some of the mutant lines. Moreover, the candidate genes, including genes encoding high-affinity nitrate transporter 2.1, gibberellin responsive protein, were identified for resistance to low N. This study raises plausible mechanistic hypotheses that can be evaluated in future applied or basic efforts by breeders or plant biologists seeking to develop new high-NUE wheat cultivars.

## Introduction

Nitrogen (N) is one of the essential elements for plant growth, and the application of N fertilizer to crops results in a dramatically increased yield [1]. However, 50% to 70% of the N fertilizer applied to production fields is not actually utilized by crops; this results in negative impacts to the environment such as the eutrophication of water supplies [2]. The improvement of nitrogen use efficiency (NUE) in crops is therefore of enormous importance [3]. Viewed in this context, the identification of any genetic loci or significant molecular markers associated with resistance to low N will be useful for the improvement of NUE in crops [4].

Wheat (*Triticum aestivum* L.) is one of the most important food crops worldwide. Genetic mapping of quantitative trait loci (QTLs) for nitrogen-related traits in wheat has been reported in previous studies [5–10]. These studies have identified QTLs for yield under a variety of different nitrogen conditions, including 12 QTLs distributed on chromosomes 1A, 2A, 2B, 2D, 3D, 4B, 5B, 6A, and 7A [8]. Another study of QTLs related to differentially responsive grain yields in response to N application identified QTLs on chromosomes 1A, 1B, 2A, 2B, 3B, 3D, 5A, 6A, 6B, 7A, and 7B, with LOD scores ranging from 3–11.8 [5]. There have also been 25 QTLs identified for differences in the value of kernel-related traits between high-N and low-N treatments on chromosomes 1B, 2A, 2B, 3B, 4B, 5D, 6B, and 7A (including 6 QTLs on chromosome 4B and 5 QTLs on chromosome 2A) [6]. It is well-established that the index of biomass and nutrient content at the seedling stage in hydroponic treatments is highly correlated with yield-related traits at the mature stage in wheat [9]. Another study reported 15 QTLs related to wheat seedling traits in response to low-N treatments on chromosomes 1B, 1D, 2B, 4A, 4B, 5D, 6A, 6B, 7A, and 7B [10].

Genome-wide association studies (GWAS) are widely used for mapping significant genetic variability from large germplasm diversity panels; such studies provide important genetic information about complex traits (i.e., traits controlled by more than one gene) in crops [11, 12]. Previous GWAS studies have implicated numerous loci with yield-related traits [13–19], disease resistance [20–23], drought resistance [24], heat tolerance [25], and so on. Several GWAS studies about nitrogen-related traits in crops have also been reported [26–28]. An association mapping study of NUE-related traits in rice identified 8 statistically significant marker loci; and two of these loci harbored known NUE-related genes [26]. A study in wheat that used 214 European winter wheat varieties identified 333 genomic regions associated with 28 NUE-related traits [27]. Additionally, a study of a global wheat core collection comprised of both land races and modern cultivars identified 23 genetic regions distributed across 16 wheat chromosomes that were putatively involved in the plant responses to different N levels [28]. Hydroponic treatment is a useful experimental approach for nutrient-responsive studies, and it is accepted that hydroponic studies can reflect strong performance for high nutrient efficiency in mature plants that are grown in field conditions [29]. However, there have been very few GWAS studies of wheat seedling traits in response to differential N treatments.

Plant genes involved in N nutrition have been studied extensively [2, 30]. Major genes for N acquisition and translocation include genes of the ammonium transporter (*AMT*) [31] and nitrate transporter (*NRT*) families [32]. It is also known that complex signaling pathways are important component for the regulation of N nutrition in plants; known signaling components include a MADS-box transcription factor [33], the *Nitrate Regulatory Gene2* (*NRG2*) [34], NIN-Like Protein 6 (*NLP6*), *NLP7* [35], and the E3 SUMO ligase (*AtSIZ1*) gene [36]. Molecular genetics studies of wheat responses to N starvation have noted differential expression patterns for *TaNRT1*, *TaNRT2* [37], and 23 different *TaAMTs* [38]. In addition, the *TaNAC* [39] and *TabHLH1* [40] transcription factors in wheat were shown to be important regulators of N signaling.

Mutagenesis has long been a centrally important experimental tool in crop improvement research programs [41]. Indeed, to date, more than 3,200 crop cultivars have been developed based on induced mutations [42]. Here, using a diversity panel comprised of 190 stable wheat mutant lines (M_6_ generation) in the Jing411 background, we identified significant SNPs associated with resistance to low N of wheat seedlings by combining GWAS and *t*-test method. This study revealed novel allelic variation and key genes affecting resistance to N limitation, particularly on chromosomes 2A and 6B, and thus provides important insights for breeders using molecular methods to improve NUE performance in wheat.

## Materials and methods

### Plant materials

The seeds of Chinese winter wheat (*Triticum aestivum* L.) cultivar Jing411 were used for EMS and γ-rays mutagenesis, and the methods for EMS and γ-rays mutagenesis were the same as previously described [43]. After phenotypic selection for several generations, 190 individual M_6_ mutant lines showing observable phenotypic changes such as plant height, flowering time, were used for genotyping and N treatment.

### Experimental design for N treatment, phenotyping, and data analysis

After germination in water for three days, the WT and mutant lines were grown in nutrient solution with either normal (4 mM) or low N (1/50 of the normal amount N) for 13 days; the composition of the nutrient solution was according to a previous study in wheat [10]. This experiment was conducted in a greenhouse (temperature 20–26°C and humidity 60%) with sunlight and two additional hours of 200–300 μmol m^-2^ s^-1^ light. For each genotype, 15 plants were treated, and finally a total of 8 plants with similar growth status from each genotype and each experimental group were sampled as replicates for data measurement. The following phenotypic values were measured for each genotype under low and normal N conditions: root length, shoot length, root weight, and shoot weight. The experiment was independently conducted twice. Based on the measured data, the relative root length (RRL), relative shoot length (RSL), relative root weight (RRW), and relative shoot weight (RSW) values were calculated according to the following formulas, respectively: RRL = Root length in low N / Root length in normal N; RSL = Shoot length in low N / Shoot length in normal N; RRW = Root weight in low N / Root weight in normal N; RSW = Shoot weight in low N / Shoot weight in normal N. The best linear unbiased estimates (BLUE) values for RRL, RSL, RRW, and RSW from 8 replicates and 2 independent experiments were used for marker-trait association.

Analyses of BLUE, variance, correlation coefficients, and broad sense heritability were performed by using the ANOVA analysis tools of the QTL IciMapping v4.1 program (http://www.isbreeding.net/).

### Genotyping and filtering

The Axiom® Wheat 660K Genotyping Array (Thermo) was used to genotype the WT and 190 mutant lines; this wheat SNP genotyping array was described in a previous study [44], and the genotyping was performed by China Golden Marker (Beijing) Biotech Co. Ltd. (CGMB, http://www.cgmb.com.cn/). Quality filtering of the genotyping data (‘pruning’) was performed using R 3.4.1 (http://www.r-project.org/). This filtration identified 463,826 SNPs that failed a ‘frequency test’ (minor allele frequency, MAF < 0.05) and 66,381 SNPs that failed a ‘missing test’ (Call-Rate < 0.97); these putative SNPs were thus excluded from further analysis. Finally, 67,402 SNP markers were used for GWAS.

### Genome-wide association study

GWAS was performed using the General Linear Model (GLM) and the Mixed Linear Model (MLM) in TASSEL 5.0 [44–46]; based on the deviation of the observed statistic values from the expected statistic values in Q-Q plots, we selected the best model MLM from the GWAS analysis of the RRL, RSL, RRW, and RSW traits. Marker-trait association examined relationships between 67,402 SNP markers and the BLUE values of RRL, RSL, RRW, and RSW trait data calculated from 8 replications and 2 independent experiments. According to the general distribution of all *p* values of the SNPs for each trait, we selected a suggestive significance threshold of *p* values ≤ 0.001 for RRL and *p* values ≤ 0.01 for RSL, RRW, and RSW. The *p* value distributions of SNPs across the chromosomes were visualized using Manhattan plots that were constructed using R. Finally, 364 SNPs were identified.

### Identification of significant SNPs by *t*-test

The identified 364 SNPs were further screened by statistically analyses of phenotypic data. The RRL, RSL, RRW and RSW from WT and mutant allele groups were compared. The phenotypic data in the two allele groups with significant difference of *p* ≤ 0.05 by *t*-test were detected as significant SNPs.

### Identification of candidate genes

By using the flanking sequencing of the significant SNP markers, a BLAST search was performed against the reference genome Chinese Spring wheat v1.0 (http://www.wheatgenome.org). The genes containing the significant SNP markers were identified as the ‘candidate genes,’ and the gene annotations were based on the BLAST searching against gene sequence from other cereal plants in NCBI (https://www.ncbi.nlm.nih.gov/).

## Results

### Assessment and correlations among wheat seedling traits related to plant resistance to low nitrogen

**Fig 1:**
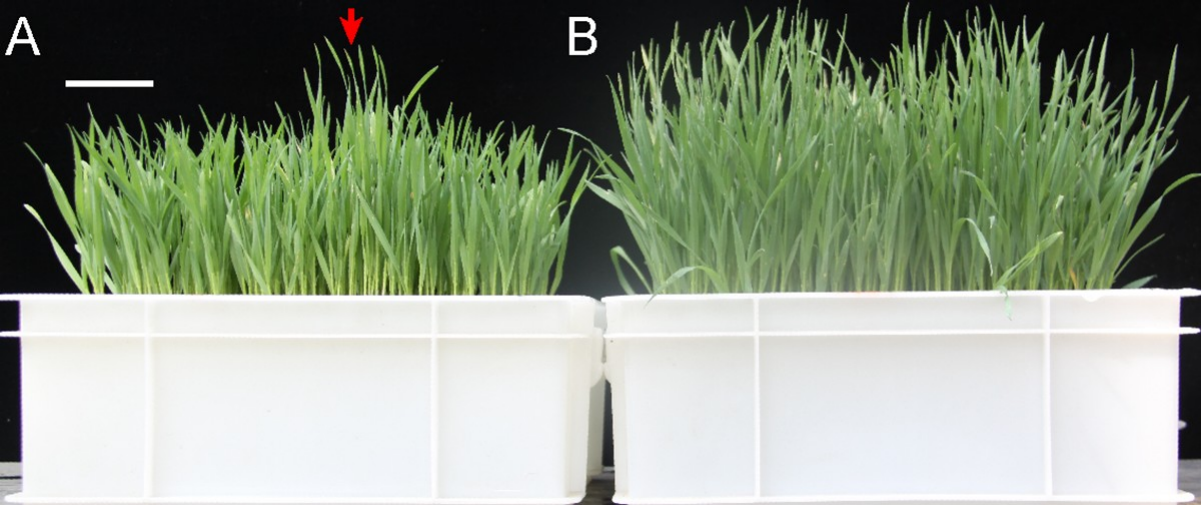
Phenotypes of wheat plants grown in low or normal N conditions. (A) WT and mutant wheat plants were grown in low-N nutrient solutions for 13 days. The red arrow indicates the wheat mutant lines that exhibited resistance to low N (*i*.*e*., taller than average lines). (B) The control WT and mutant plants grown under normal N condition. Bar = 5 cm.

Plants of the M_6_ generation of 190 wheat mutant lines from the mutant library of Jing411 background, which showed observable phenotypic changes (eg. plant height, flowering time), were grown hydroponically with either normal or low N concentrations. Unsurprisingly, wheat seedlings grown in the low-N treatment were much smaller than the seedlings grown with normal N treatment. Some of the mutants showed resistance to the low-N treatment (as evidence by taller growth stature and/or increased fresh weight) (Fig 1). The relative root length (RRL), relative shoot length (RSL), relative root weight (RRW), and relative shoot weight (RSW) were calculated and used as indices for resistance to the low N treatment. The mean values of RRL, RSL, RRW, and RSW in WT and mutants of two independent experiments were shown in S1 Table. Among the 190 mutant lines, 5 lines showed higher relative shoot length and shoot weight (more than 26% higher than that of WT in both experiments), indicating the effects of low N treatment on the shoot growth of these lines were lower. Therefore, these mutants were considered as resistance to the low N treatment. ANOVA analysis indicated that the variance among the WT and the 190 mutant genotypes for all four investigated traits (in two independent experiments) were significant at the *p* ≤ 0.05 level (Table 1). Further, Pearson correlation coefficient analysis between analytical pairings of each of the traits ranged from 0.52 to 0.77 (RRL, RSL, RRW, and RSW) (Table 2), indicating positive correlations among these phenotypic traits. Best linear unbiased estimates (BLUE) value analysis showed that variation ranged from 0.93 to 2.61 for RRL, from 0.55 to 1.18 for RSL, from 0.68 to 2.28 for RRW, and from 0.12 to 1.06 for RSW; the coefficient of variation for the four traits ranged from 12.75–20.22% (Table 3), highlighting wide variation for each trait among the different wheat mutant lines. Further, each of these four phenotypic traits related to resistance to low N exhibited high broad sense heritability, with *H*^*2*^ values ranging from 0.48–0.71.

**Table 1:** Analysis of variance for the investigated traits.

Trait	Genotype	Experiment
RRL	10.9368***	3.8036*
RSL	15.1476***	286.9342***
RRW	3.381***	9.4928**
RSW	4.1495***	22.068***

The following traits were investigated in this study: the relative root length (RRL), relative shoot length (RSL), relative root weight (RRW), and relative shoot weight (RSW).

* indicates statistical significance at *p* ≤ 0.05

** indicates statistical significance at *p* ≤ 0.01

*** indicates statistical significance at *p* ≤ 0.001.

**Table 2:** Correlation coefficients among the four phenotypic traits.

Trait	RSL	RRL	RSW
RRL	0.52***		
RSW	0.77***	0.56***	
RRW	0.57***	0.72***	0.65***

*** Statistical significance at *p* ≤ 0.001.

**Table 3:** Summary statistics for the four phenotypic traits among the WT and mutant lines.

Trait	RRL	RSL	RRW	RSW
Mean	1.31	0.73	1.21	0.63
Minimum	0.93	0.55	0.68	0.12
Maximum	2.61	1.18	2.28	1.06
SD	0.23	0.09	0.25	0.12
CV%	17.28	12.75	20.22	19.41
*H*^*2*^	0.69	0.71	0.48	0.50

SD, standard deviation; CV, coefficients of variation; *H*^*2*^, broad sense heritability.

### The SNPs loci associated with seedling resistance to low N by MLM analysis

To obtain reliable marker-trait associations, we based the analysis on BLUE values for the four traits from two independent experiments. Based on the deviation of the observed statistic values from the expected statistic values in the Q-Q plots, we determined that a MLM model was superior to a GLM model for GWAS of RRL, RSL, RRW, and RSW (S1 Fig). Finally, a total of 364 SNPs were detected for association with RRL, RSL, RRW, and RSW.

**Fig 2:**
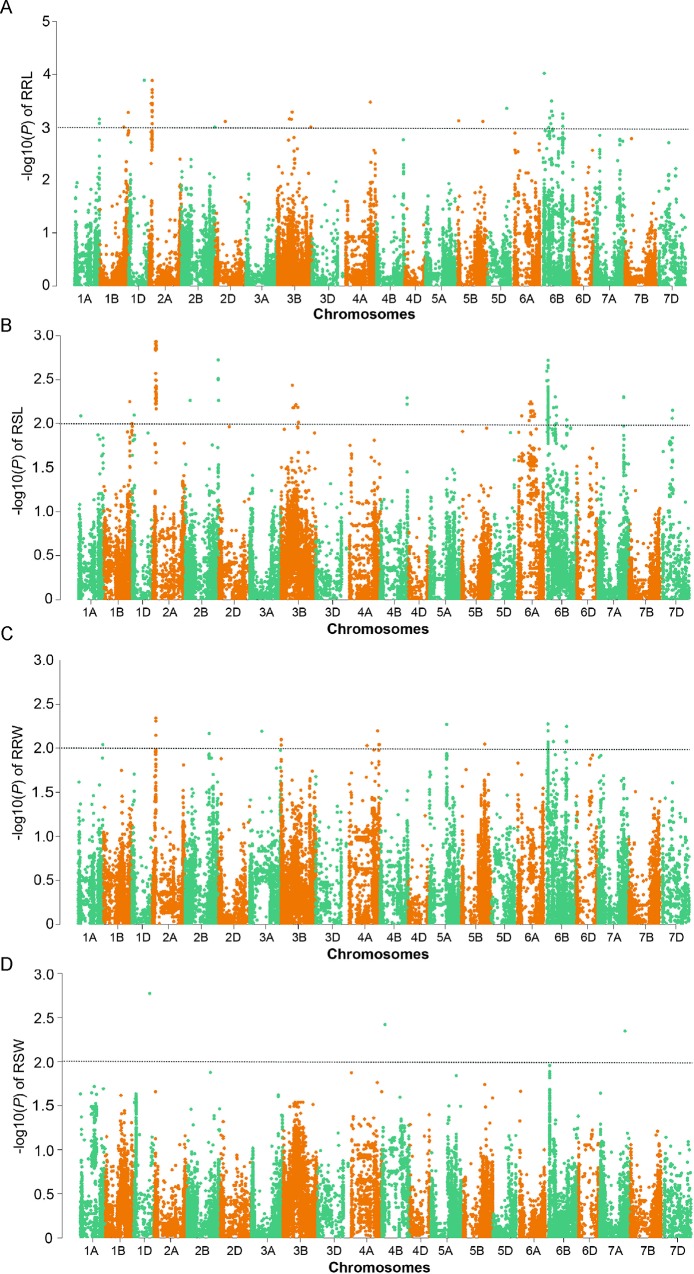
Manhattan plots of the four phenotypic traits. (A) RRL; (B) RSL; (C) RRW; (D) RSW. The black dashed lines indicate–log_10_ transforms of the suggestive *p* values.

For RRL, a total of 95 SNPs, distributed on chromosomes 1A, 1B, 1D, 2A, 2B, 2D, 3B, 4A, 5B, 5D, and 6B, were identified at the *p* ≤ 0.001 level (S2 Table). Among these, the highest number of SNPs was detected on chromosome 2A (Table 4 and Fig 2A). Strikingly, fully 10% of the total phenotypic variation for RRL could be explained by marker AX-111453204 on chromosome 2A (Table 4). Additionally, on chromosome 6B, 19 significant SNPs were detected, and the marker AX-110666921 also account for 10% of the phenotypic variation for RRL (Table 4).

**Table 4:** List of markers associated with RRL, RSL, RRW, and RSW by GWAS.

Trait	Marker	SNP	Chr.	Position	*P*-value	R^2^
RRL	AX-110941028	2	1A	587818235–588491731	6.95E-04	0.08
	AX-108868526	2	1B	574569218–675535984	5.24E-04	0.08
	AX-108811005	1	1D	372812518	1.29E-04	0.10
	AX-111453204	60	2A	58538037–77335120	1.31E-04	0.10
	AX-111611740	1	2B	793179533	9.94E-04	0.08
	AX-110467352	1	2D	258384771	7.76E-04	0.08
	AX-109951689	5	3B	283539431–800088745	5.19E-04	0.08
	AX-89472629	1	4A	595265943	3.38E-04	0.07
	AX-109031151	2	5B	29569824–606299220	7.59E-04	0.08
	AX-111358076	1	5D	458076450	4.41E-04	0.09
	AX-110666921	19	6B	25806926–476960396	9.61E-05	0.10
RSL	AX-110618032	1	1A	53447083	8.20E-03	0.05
	AX-110376696	2	1B	629148059–629151382	5.62E-03	0.04
	AX-94723578	1	1D	46631942	7.98E-03	0.05
	AX-108805657	103	2A	75143409–77335120	1.18E-03	0.06
	AX-110431432	5	2B	111285723–793179533	1.90E-03	0.07
	AX-111638430	7	3B	261150728–413095187	3.66E-03	0.06
	AX-111039474	2	4B	643481153–643501106	5.12E-03	0.06
	AX-111490103	18	6A	104119755–405861085	5.68E-03	0.06
	AX-111450720	85	6B	2447095–476960396	1.91E-03	0.07
	AX-110475574	2	7A	610616256–610872015	4.97E-03	0.06
	AX-110829820	3	7D	231185616–239849103	7.06E-03	0.04
RRW	AX-110941028	1	1A	587818235	9.06E-03	0.05
	AX-111022688	6	2A	77267434–77335120	4.58E-03	0.04
	AX-109979330	1	2B	574720198	6.78E-03	0.04
	AX-109915920	1	3A	287684775	6.41E-03	0.05
	AX-110125256	3	3B	667972–2235874	7.95E-03	0.05
	AX-108888540	4	4A	427992360–723204067	6.34E-03	0.05
	AX-109984948	1	5A	409357352	5.34E-03	0.04
	AX-110913918	1	5B	559445291	8.97E-03	0.04
	AX-110666921	19	6B	25806926–476042649	5.31E-03	0.06
RSW	AX-108811005	1	1D	372812518	1.69E-03	0.07
	AX-111456285	1	4B	68900101	3.81E-03	0.06
	AX-111539752	1	7A	603542835	4.52E-03	0.06

For each phenotypic trait, the total numbers and the range of positions of the SNPs distributed across the chromosomes are given. The SNPs showing the lowest *p* value for each chromosome are listed with marker names, *p*-value, and R^2^.

For RSL, a total of 229 markers were detected at the *p* ≤ 0.01 level, and these were distributed on chromosomes 1A, 1B, 1D, 2A, 2B, 3B, 4B, 6A, 6B, 7A, and 7D (S3 Table). As with RRL, many of these SNPs (103) were located on chromosome 2A and many (85) were on chromosome 6B (Table 4 and Fig 2B). The SNP marker AX-108805657 on chromosome 2A and the marker AX-111450720 on chromosome 6B explained, respectively, 6% and 7% of the phenotypic variation for RSL (Table 4).

There were relatively fewer SNPs at the *p* ≤ 0.01 level for RRW: 37 SNPs (S4 Table), distributed on chromosomes 1A, 2A, 2B, 3A, 3B, 4A, 5A, 5B, and 6B, were associated with RRW at the *p* ≤ 0.01 level (Table 4 and Fig 2C). Fully half of these SNPs for RRW were located on chromosome 6B, and the AX-110666921 marker explained 6% of the phenotypic variation for RRW (Table 4). Finally, there were only 3 SNPs for RSW at the *p* ≤ 0.01 level (S5 Table) distributed on chromosomes 1D, 4B, and 7A, explained 7%, 6%, and 6% of the phenotypic variation, respectively (Table 4 and Fig 2D).

### The significant SNP loci resulted in phenotypic variations between WT and mutants by *t*-test

We further investigated the significant SNPs among the detected 364 SNPs, which statistically resulted in phenotypic variations by *t*-test. Finally, a total of 221 SNPs significantly increased the RRL, RSL, and RRW, respectively, in the allele of mutant group compared to that of WT group (S6 Table). Generally, the number of lines with mutant allele ranging from 7 to 88 was observed among the significant SNP loci. For RRL, two SNPs were distributed on chromosomes 1A and 6B, respectively. For RSL, the significant SNPs were detected on chromosomes 2A, 2B, 4B, 6A, 6B, and 7A, including 103 and 81 SNPs on chromosome 2A and 6B, respectively. For RRW, the significant SNPs were distributed on chromosomes 2A, 2B, 4A, 5A, and 6B. Additionally, 6 significant SNPs (marker AX-110666921, AX-111453204, AX-110574568, AX-111023022, AX-110530579, AX-95011058) were associated with two of the three traits.

### Candidate genes associated with resistance to low N

Genes containing the significant SNPs that resulted in statistically variation of RRL, RSL, RRW, and RSW in the mutant allele would be important for resistance to low N and were further examined in this study. A total of 41 SNPs occurred in genic sequences, including 19 on chromosome 6B and 22 on chromosome 2A (Table 5). BLAST-based annotation of these candidate genes suggested that 1 significant SNP (AX-94852973, mutation in 71 lines) resulted in amino acid change of a gene encoding high-affinity nitrate transporter 2.1, and another significant SNP (AX-95011058, mutation in 88 lines) occurred in a gene encoding gibberellin responsive protein; 11 significant SNPs occurred in three genes encoding disease resistance protein RPP13-like; 2 SNPs occurred in a gene encoding UDP-N-acetylglucosamine—dolichyl-phosphate N-acetylglucosaminephosphotransferase-like, RNA pseudouridine synthase 6, DEAD-box ATP-dependent RNA helicase 10, respectively; and 3 SNPs occurred in two genes encoding L-type lectin-domain containing receptor kinase. Additionally, the significant SNPs were also observed in a gene involving in bifunctional protein-serine/threonine kinase/phosphatase, transcription termination factor MTERF15, pre-mRNA-processing factor 39-like, protein STRUBBELIG-RECEPTOR FAMILY 5-like, UPF0481 protein At3g47200-like, G-type lectin S-receptor-like serine/threonine-protein kinase, ABC transporter C family member 10-like, and cis-zeatin O-glucosyltransferase 1-like (Table 5).

**Table 5:** The candidate genes associated with resistance to low N.

Marker	Gene ID	Gene annotation
AX-94852973	TraesCS6B01G044000	high-affinity nitrate transporter 2.1-like
AX-95011058	TraesCS6B01G050700	gibberellin responsive protein gene
AX-109488831	Traes_6BS_6C812D341	disease resistance protein RPP13-like
AX-108831423	Traes_6BS_6C812D341	disease resistance protein RPP13-like
AX-111068223	Traes_6BS_6C812D341	disease resistance protein RPP13-like
AX-110666921	TraesCS6B01G041800	disease resistance RPP13-like protein 3
AX-111581633	TraesCS6B01G041800	disease resistance RPP13-like protein 3
AX-108891336	TraesCS6B01G041800	disease resistance RPP13-like protein 3
AX-109404235	TraesCS6B01G041800	disease resistance RPP13-like protein 3
AX-111078323	TraesCS6B01G041800	disease resistance RPP13-like protein 3
AX-89334353	TraesCS6B01G041800	disease resistance RPP13-like protein 3
AX-110475152	TraesCS6B01G041800	disease resistance RPP13-like protein 3
AX-109464253	TraesCS6B01G043500	disease resistance protein RPP13-like
AX-111518118	Traes_6BS_143FEF476	Bifunctional protein-serine/threonine kinase/phosphatase
AX-111175044	Traes_6BS_A5FB0FF9D	transcription termination factor MTERF15
AX-110071044	Traes_6BS_FF3A794F8	pre-mRNA-processing factor 39-like
AX-108918819	TraesCS6B01G051000	protein STRUBBELIG-RECEPTOR FAMILY 5-like
AX-111450720	Traes_6BS_26326DD6C	UPF0481 protein At3g47200-like
AX-110544749	Traes_2AS_8DF7D907F	G-type lectin S-receptor-like serine/threonine-protein kinase
AX-109010730	Traes_2AS_1CBBCBAA2	ABC transporter C family member 10-like
AX-108805657	Traes_2AS_FB8C94F8E	UDP-N-acetylglucosamine—dolichyl-phosphate N-acetylglucosaminephosphotransferase-like
AX-111519665	Traes_2AS_FB8C94F8E	UDP-N-acetylglucosamine—dolichyl-phosphate N-acetylglucosaminephosphotransferase-like
AX-110360537	Traes_2AS_C3CF1C55F	RNA pseudouridine synthase 6, chloroplastic
AX-110428187	Traes_2AS_C3CF1C55F	RNA pseudouridine synthase 6, chloroplastic
AX-111040684	Traes_2AS_617EE2FA7	DEAD-box ATP-dependent RNA helicase 10
AX-110396519	Traes_2AS_617EE2FA7	DEAD-box ATP-dependent RNA helicase 10
AX-111749124	Traes_2AS_1FA413046	L-type lectin-domain containing receptor kinase IV.1-like
AX-111465844	Traes_2AS_1FA413046	L-type lectin-domain containing receptor kinase IV.1-like
AX-109345931	Traes_2AS_B834BD325	L-type lectin-domain containing receptor kinase IV.1-like
AX-110365502	TraesCS2A01G128200	cis-zeatin O-glucosyltransferase 1-like
AX-109973857	TraesCS2A01G127800	uncharacterized protein
AX-110609989	TraesCS2A01G127800	uncharacterized protein
AX-110652048	TraesCS2A01G128400	uncharacterized protein
AX-110907563	TraesCS2A01G127800	uncharacterized protein
AX-109833412	Traes_2AS_4ACFB257F	uncharacterized protein
AX-110383858	Traes_2AS_4ACFB257F	uncharacterized protein
AX-108939488	TraesCS2A01G130100LC	uncharacterized protein
AX-109419490	TraesCS6B01G194500	uncharacterized protein
AX-110504653	Traes_2AS_42C13EE1C	uncharacterized protein
AX-111715384	Traes_2AS_42C13EE1C	uncharacterized protein
AX-108770012	Traes_2AS_42C13EE1C	uncharacterized protein

Genes containing the significant markers are identified as the candidate gene and listed.

## Discussion

Understanding the genetic basis of resistance to low N in crops is an important building block for NUE improvement strategies [3]. In this study, using a population derived from induced mutagenesis in wheat, we characterized allelic variation that affects seedling resistance to low N. Induced mutagenesis methods reliably produce large numbers of genetic and thus phenotypic variations [47, 48]. Compared to the diploid species Arabidopsis, treatment of hexaploid wheat with common mutagens results in considerably higher mutation frequencies (~one mutation per 30 kb) [42]. The combining of the MLM analysis and *t*-test of phenotypic traits in the wheat mutant population for identification of the significant SNPs provides an effective route for investigation the novel SNP loci and/or genes in resistance to low N.

N use efficiency is tightly connected with the agronomic traits such as plant height and flowering time [49]. In this study, we used 190 mutant lines showing observable phenotypic changes (eg. plant height, flowering time) for genotyping and N treatment. It is reasonable to speculate that more genomic variations exist in these mutants. Interestingly, we found that the phenotypic data for four traits for resistance to low N were highly variable among the 190 mutant lines (Tables 1 and 3). Hydroponic methods are often used in studies of nutrient metabolism and signaling in plants, because they are relatively easy to use and enable very precise control of nutrient delivery. Importantly, it has been reported that the nutrient-related traits observed in hydroponic system in seedling-stage plants are significantly positively correlated to N and P uptake efficiency traits monitored for mature plants grown in field conditions [9, 29]. The four traits measured in this study (RRL, RSL, RRW, and RSW) reflect the NUE levels. We observed mutant wheat lines with relatively higher seedling length and/or fresh weights under low-N treatment (Fig 1 and S1 Table), indicating the potential of identifying high-NUE performers from induced mutagenesis populations. Obviously, the detailed mechanisms underlying the observed resistance to low N, and any practical application of the mutant lines of interest under the field condition will require further characterization in future studies.

Although there have been few GWAS of NUE in wheat, the limited information available suggests that the almost all chromosomes have at least some regions that affect NUE [27, 28]. In our study, the loci associated with the four resistance to low N traits were located on 17 chromosomes; that is, all chromosomes excepting 3D, 4D, 6D, and 7B (Table 4 and Fig 2). By using mutant population in this study, the allele could be easily classified into two groups (WT and mutant allele groups). Therefore, the changes of phenotypic data resulted from allele variation would be statistically detected by *t*-test. The combining of GWAS and *t*-test restricted the significant markers associated with resistance in low N to chromosome 1A, 2A, 2B, 4A, 4B, 5A, 6A, 6B, 7A, and most of the significant SNPs were located on chromosome 2A and 6B (S6 Table). Previous QTL mapping studies of wheat grain yield in response to varying N application indicated that a QTL on chromosome 2A explained a high proportion of phenotypic variance as evaluated across three field test sites [5]. This is consistent with our finding that the highest number of significant SNPs associated with RSL was observed on chromosome 2A (S6 Table). Additionally, a QTL study of kernel-related traits in plants grown under different N conditions also identified multiple QTLs on chromosome 2A [6]. The chromosome 6B also exhibited higher amounts of significant SNPs associated with RSL and RRW (S6 Table). Meanwhile, the genomic regions that were associated with more than one of the four traits suggested that loci on chromosomes 2A and 6B were significantly associated with RRL, RSL, and RRW (Fig 2 and Table 4). These results clearly suggested that these regions appear to somehow confer resistance to low N. Previous QTL mapping studies of seedling traits related to N nutrition also identified significant QTLs on chromosome 6B, but did not report QTLs for chromosome 2A [9, 10].

The mutated genes resulting in relative phenotypic data variations under low N to normal N condition would be important for resistance to low N. Interestingly, 1 significant SNP occurred in a gene encoding high-affinity nitrate transporter 2.1 (NRT2.1) and the mutation was found in 71 lines (S6 Table and Table 5). It is well documented that NRT1 and NRT2 family transporters mediate nitrate uptake from soil [32] and the Arabidopsis NRT2.1 play a central role in coordinating root response to N limitation [50]. Moreover, it has been suggested that the transcript level of wheat NRT2.1 was significantly induced by N starvation [37]. In this study, the mutation of NRT2.1 in the 71 lines leaded to the encoded amino acid changes at the site of 402, which probably resulted in the phenotypic variation in response to low N. Gibberellins are essential regulators for plant development and closely related to N acquisition in plant [51]. It has been suggested that GA signaling pathway participated in regulation of N deficiency-induced anthocyanin accumulation [52]. Conversely, N availability modulates the activity of GA transporter NPF3.1 in Arabidopsis [53]. Therefore, it is reasonable to observe that the mutation of gibberellin responsive protein gene resulted in resistance to low N compared to that of WT- allele group (S6 Table and Table 5). Additionally, 11 significant SNPs occurred in three genes encoding disease resistance protein RPP13-like, suggesting the important roles of RPP13 genes for resistance in low N. Disease resistance proteins are a well-known large family of proteins that are important in the regulation of plant disease resistance responses [54, 55]. A previous study showed that expression of the gene encoding a disease resistance protein was differentially regulated by different forms of N [56]. Clearly, the possible low N resistance function of the candidate disease resistance protein RPP13-like identified in the present study will require further investigation.

## Conclusions

We here identified SNPs that were significantly association with the low N resistance traits RRL, RSL, RRW, and RSW in wheat by combining GWAS and *t*-test methods. Of particular note, loci on chromosomes 2A and 6B were found to be especially impactful for resistance to low N. Several candidate genes, including genes encoding a high-affinity nitrate transporter 2.1 and a gibberellin responsive protein, were implicated as having possible functions associated with resistance to low N. Future work can validate the significant markers we identified here and can determine whether or not any of these markers will be effective in NUE-improvement efforts in wheat. Finally, this study deepens our knowledge about the genetic basis of a core metabolic nexus in arguably the world’s most important food crop.

## Supporting information

S1 Fig Comparison of Q-Q plots for RRL using GLM (A) and MLM (B). The black line indicates the expected values.
(TIF)

S1 Table The mean values of RRL, RSL, RRW, and RSW in WT and mutants of two independent experiments. The five mutant lines with bold font showing higher RSL and RSW in both experiments were considered as resistance to the low N treatment. The 1^st^ or 2^nd^ in the brackets indicate the values in the column are from the first or the second experiment.
(XLSX)

S2 Table The SNP markers associated with RRL at *p* ≤ 0.001 by GWAS. (XLSX)

S3 Table The SNP markers associated with RSL at *p* ≤ 0.01 by GWAS. (XLSX)

S4 Table The SNP markers associated with RRW at *p* ≤ 0.01 by GWAS. (XLSX)

S5 Table The SNP markers associated with RSW at *p* ≤ 0.01 by GWAS. (XLSX)

S6 Table The significant SNPs associated with the traits related to low N resistance by t-test. ^a, b^ indicate the statistically significant difference of the traits in two allele groups by *t*-test; ^c, e^ represent the allele in WT and mutant, respectively; ^d, f^ show the mean values and SD of RRL, RSL and RRW in the WT and mutant allele groups, respectively.
(XLSX)
