# Supplementary figures and images for: A combined association mapping and t-test analysis of SNP loci and candidate genes involving in resistance to low nitrogen traits by a wheat mutant population
Source: PLoS One. 2019 Jan 30;14(1):e0211492. doi: 10.1371/journal.pone.0211492 (PMC6353201; doi:10.1371/journal.pone.0211492)

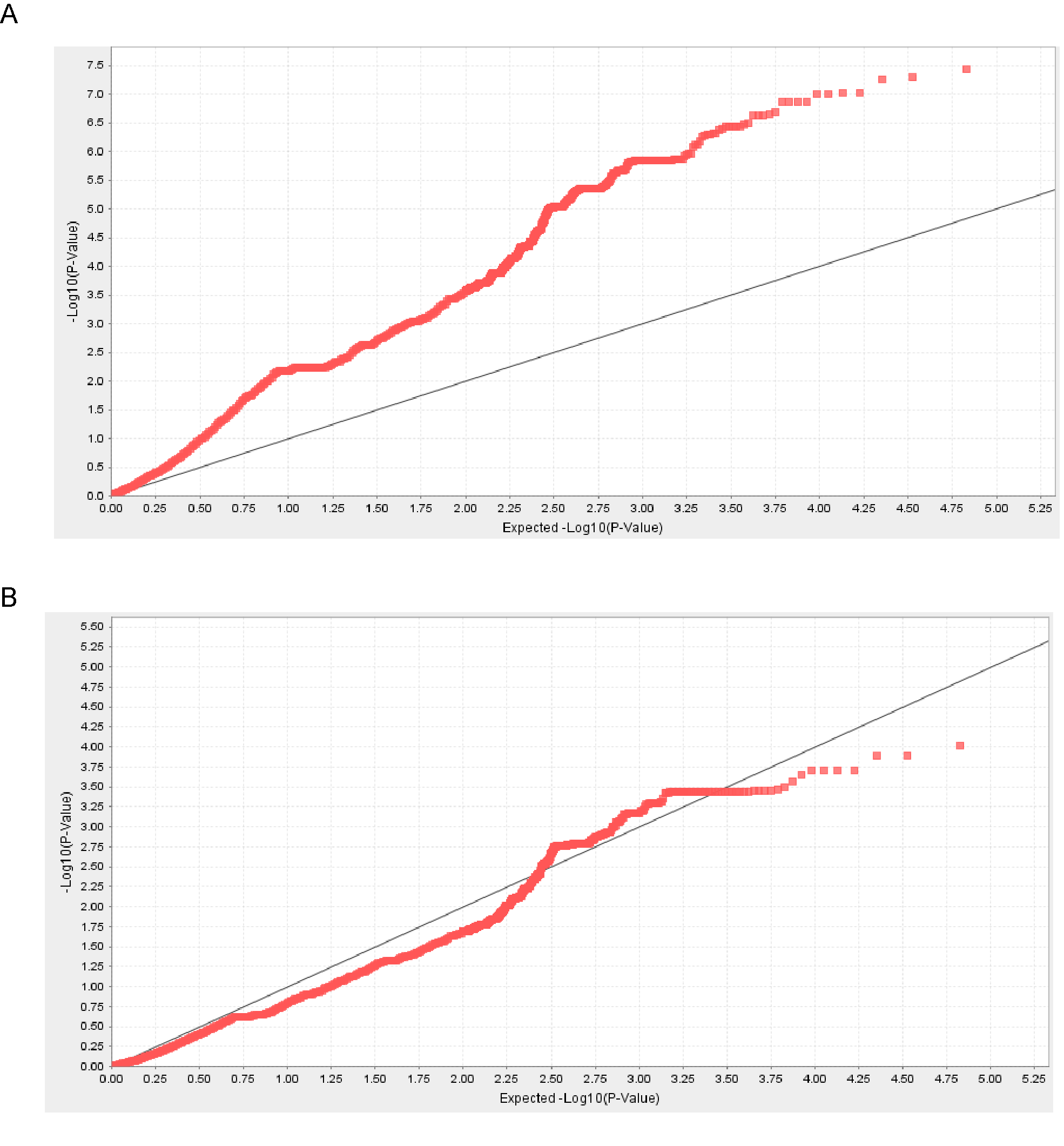

Supplement: S1 Fig — Comparison of Q-Q plots for RRL using GLM (A) and MLM (B). The black line indicates the expected values. (TIF) [file pone.0211492.s001.tif]
